# PSYCHOSOCIAL CARE AFTER STROKE AS PROVIDED BY MULTIDISCIPLINARY TEAMS IN STROKE SERVICES

**DOI:** 10.1093/geroni/igad104.0672

**Published:** 2023-12-21

**Authors:** Dagmar van Nimwegen, Lisette Schoonhoven, Johanna Visser-Meily, Janneke M de Man-van de van de van Ginkel

**Affiliations:** University of Applied Sciences Utrecht, Utrecht, Utrecht, Netherlands; University Medical Center Utrecht, Utrecht University, Utrecht, Utrecht, Netherlands; UMCU, Utrecht, Utrecht, Netherlands; Leiden University Medical Center, Leiden, Zuid-Holland, Netherlands

## Abstract

About 18-31% of all persons with stroke develop one or more psychosocial impairments, such as depression or anxiety. They receive care in stroke services from multidisciplinary teams, consisting of several different healthcare professionals. Currently, it is unclear how psychosocial care is provided within these stroke services, what roles healthcare professionals play in this, and what psychosocial needs persons with stroke experience. Therefore, in this study the following research questions were answered: How do persons with stroke and healthcare professionals experience the current psychosocial care after stroke, and what psychosocial needs do persons with stroke experience? A qualitative study was conducted, which consisted of observations of care moments between 44 persons with stroke and 74 healthcare professionals, and individual interviews with ten persons with stroke. The observations showed that most care moments mainly focus on physical care. Healthcare professionals tend to focus on the care they give at that particular moment. In contrast, persons with stroke asked attention for a broader perspective on the whole rehabilitation process. In addition, the interviews showed that persons with stroke mostly need: clarity about the rehabilitation process, the ability to make their own decisions, a personal approach in stroke care, and to recover as fast as possible. To be able to provide care that fulfills the needs of persons with stroke, awareness about the differences in perspective between healthcare professionals and patients is essential in care programs, possibly leading to the necessity to change such programs to better fit these needs.

